# Supplementary material for: DnoisE: distance denoising by entropy. An open-source parallelizable alternative for denoising sequence datasets
Source: PeerJ. 2022 Jan 19;10:e12758. doi: 10.7717/peerj.12758 (PMC8783565; doi:10.7717/peerj.12758)
Supplement: Supplementary file 1 [file peerj-10-12758-s003.odt]

Supplementary materials:

Scripts for running the comparison of DnoisE and Unoise3 and to evaluate the performance of both programs.

run_unoise.sh

| #!/bin/bash input_file=/PATH/TO/FILE output_dir=/PATH/TO/DIRECTORY/ # Unoise3 /home/soft/usearch-10.0/usearch10.0.240 -unoise3 ${input_file} -zotus ${output_dir}zotus.fasta -tabbedout ${output_dir}out.txt -ampout ${output_dir}out.fasta -unoise_alpha 5 -minsize 1 |
| --- |

run_DnoisE_Xc_Xj.sh

| #!/bin/bash input_file=/PATH/TO/FILE output_dir=/PATH/TO/DIRECTORY/ scripts_dir=/PATH/TO/DnoisE/src/ cores=<number_of_threads> output_type=< 1 │ 2 │ 3 │ 4 > # DnoisE without entropy correction # it will run with ${cores} threads and the output joining criteria is set with ${output_type} ${scripts_dir}DnoisE --fasta_input ${input_file} --csv_output ${output_dir}output -c ${cores} -j ${output_type} 2>${output_dir}${cores}_progress |
| --- |

run_DnoisE_Xc_Xj_corr.sh

| #!/bin/bash input_file=/PATH/TO/FILE output_dir=/PATH/TO/DIRECTORY/ scripts_dir=/PATH/TO/DnoisE/src/ cores=<number_of_threads> output_type=< 1 │ 2 │ 3 │ 4 > # DnoisE with entropy correction and entropy values set as 0.473,0.227,1.021 # it will run with ${cores} threads and the output joining criteria is set with ${output_type} ${scripts_dir}DnoisE --fasta_input ${input_file} --csv_output ${output_dir}output -c ${cores} -j ${output_type} -y -e 0.473,0.227,1.021 2>${output_dir}${cores}_progress |
| --- |

script_to_get_time_and_memory_usage.sh

| #!/bin/bash # in order to get the time and memory usage, run the following command # cores and output type must be specified in the run_DnoisE_Xc_Xj.sh or run_DnoisE_Xc_Xj_corr.sh script=/PATH/TO/SCRIPT/run_DnoisE_Xc_Xj.sh # or script=/PATH/TO/SCRIPT/run_DnoisE_Xc_Xj_corr.sh if entropy correction is performed /usr/bin/time -f "Wall clock time: %E\nMaximum size (Kb): %M\n" bash ${script} 2>store_values.txt script_unoise=/PATH/TO/SCRIPT/run_unoise.sh /usr/bin/time -f "Wall clock time: %E\nMaximum size (Kb): %M\n" bash ${script_unoise} 2>store_values_unoise.txt |
| --- |
